# An Enhanced Data Visualization Method for Diesel Engine Malfunction Classification Using Multi-Sensor Signals

**DOI:** 10.3390/s151026675

**Published:** 2015-10-21

**Authors:** Yiqing Li, Yu Wang, Yanyang Zi, Mingquan Zhang

**Affiliations:** State Key Laboratory for Manufacturing Systems Engineering, School of Mechanical Engineering, Xi’an Jiaotong University, No. 28 Xianning West Road, Xi’an 710049, China; E-Mails: vikinimo@gmail.com (Y.L.); ziyy@mail.xjtu.edu.cn (Y.Z.); zmqf117@stu.xjtu.edu.cn (M.Z.)

**Keywords:** multi-sensor signals, data visualization, feature subset score, diesel engine, malfunction classification

## Abstract

The various multi-sensor signal features from a diesel engine constitute a complex high-dimensional dataset. The non-linear dimensionality reduction method, t-distributed stochastic neighbor embedding (t-SNE), provides an effective way to implement data visualization for complex high-dimensional data. However, irrelevant features can deteriorate the performance of data visualization, and thus, should be eliminated *a priori*. This paper proposes a feature subset score based t-SNE (FSS-t-SNE) data visualization method to deal with the high-dimensional data that are collected from multi-sensor signals. In this method, the optimal feature subset is constructed by a feature subset score criterion. Then the high-dimensional data are visualized in 2-dimension space. According to the UCI dataset test, FSS-t-SNE can effectively improve the classification accuracy. An experiment was performed with a large power marine diesel engine to validate the proposed method for diesel engine malfunction classification. Multi-sensor signals were collected by a cylinder vibration sensor and a cylinder pressure sensor. Compared with other conventional data visualization methods, the proposed method shows good visualization performance and high classification accuracy in multi-malfunction classification of a diesel engine.

## 1. Introduction

Condition monitoring on diesel engines is essential for their safety and reliability. In the condition monitoring system of diesel engines, reliable detection and classification of engine malfunctions is very important to schedule maintenance [1]. However, the ambiguity between different malfunctions is still the main challenge in any condition monitoring system. In order to obtain more information from such a complex system, different measurement methods and condition monitoring approaches with multi-sensor systems were proposed [2,3]. Therefore, an efficient approach for classifying the malfunctions of diesel engines from multi-sensor signals is the main task of a condition monitoring system.

In recent years, different measurement methods were used for condition monitoring and diagnosis of diesel engines. These measurement methods, including vibrations [4,5,6,7], instantaneous speed [8,9,10], oil analysis [11], acoustic emission [12,13], cylinder pressure [14,15,16], *etc.*, have shown good performance in condition monitoring of the diesel engine. Of these signals, cylinder vibration signals are easy to obtain, and cylinder pressure signals can reflect the real combustion conditions of diesel engines. Therefore, these two measurement methods have been widely used in diesel engine condition monitoring systems.

However, due to the complexity of the diesel engine, it is difficult to identify the conditions or malfunctions by a single feature from one measurement signal. For example, the peak value of cylinder pressure can reflect the maximum pressure of the combustion. However, the combustion condition cannot be determined by only this parameter due to the fact that the whole combustion condition is also determined by the mean value of pressure, the maximum rate of pressure rise, the amplitude of combustion vibration, *etc*. To better reflect the engine’s health states, multiple features should be extracted from the signals of different sensors. Multi-objective optimization method and data fusion are effective ways to address the multi-sensor problem. Many works focused on multi-objective optimization algorithms and effective solutions for multi-sensor and sensor networks were proposed [17,18,19,20]. In [21], the multi-sensor data fusion method was integrated in the integrated vehicle health maintenance system (IVHMS) for reducing failure risk.

The multiple features extracted from the multi-sensor signals construct a high-dimensional dataset, which are accompanied by a large volume of data due to the continuous sampling in an online condition system. As advances in handling big and complex data, a number of methods were proposed. In these methods, data visualization can provide a way to handle big and high-dimensional data as it offers an intuitive interface for humans to rapidly detect the structural elements of the data such as clusters, homogeneous regions, outliers, *etc.* [22].

In the domain of data visualization, dimensionality reduction is one of the most common techniques. Dimensionality reduction can be used to reduce the complexity and dimension of the original data while keeping most of the desired intrinsic information [23,24]. Such intrinsic information can be used for distinguishing the different health states of tested system from a visualization perspective.

Various methods such as principal component analysis (PCA) [25] and linear discriminant analysis (LDA) [26,27] for dimensionality reduction were proposed in the literature. These methods are linear techniques that focus on keeping the low-dimensional representations of dissimilar data points far apart. Sometimes, it is more important to keep the data points of the same class close together in the low-dimensional space, especially when they have a heterogeneous distribution in high-dimensional space. It is typically not possible to use a linear mapping method, and a non-linear manifold can be more competent to do this [28].

A variety of non-linear dimensionality reduction methods such as isometric mapping (ISOMAP) [23], locally linear embedding (LLE) [24], kernel PCA [29], t-distributed stochastic neighbor embedding (t-SNE) [28] have also emerged. These methods enable the correct visualization of data which lie on curved manifolds or incorporate cluster of complex shape. In addition, most of the non-linear dimensionality reduction methods provide a map of the given data points only, without an explicit mapping prescription. This choice has a benefit that it equips the methods with a high degree of flexibility for keeping the correlations of the different classes in classification problems.

As mentioned above, the high-dimensional data which are used for data visualization contain many different features that are extracted from multi-sensor signals. For a classification problem, however, not all the features are useful for classifying. Irrelevant features may even deteriorate the classification result. In our study, in order to reflect the different malfunctions of a diesel engine, the different features are extracted from vibration signals and pressure signals. Not all the features are sensitive for the specific malfunction. When all of the features are used for dimensionality reduction, the irrelevant or non-sensitive features may deteriorate the performance of the classification in low-dimensional space. Some malfunctions which are very similar in high-dimensional space may not be separated in low-dimensional space. Furthermore, the high-dimensional data contains irrelevant features causing higher computational cost. To solve these problems, a feature subset score based t-SNE data visualization method is proposed in this paper for the malfunction classification of diesel engines. The optimal feature subset is obtained by a subset-level score criterion through an iterative algorithm. After that, it is used for data visualization by t-SNE method.

The contributions of this paper can be summarized as: (1) the data visualization method is extended to data classification using multi-sensor data; (2) according to a feature subset score criterion, an improved t-SNE algorithm, referred to as FSS-t-SNE, is proposed for data visualization. The proposed method selects the optimal features, by which the irrelevant features are eliminated; (3) the proposed method is applied on the malfunction classification of diesel engines using multi-sensor signals. In this study, the datasets from the UCI machine learning repository are used to validate the proposed method. The results show that it can effectively improve classification accuracy. Finally, a malfunction classification experiment on a diesel engine was performed to validate the proposed method. In this experiment, a 16-cylinder marine diesel engine was used. A pressure sensor and a vibration sensor were used to collect the cylinder pressure signals and cylinder vibration signals, respectively. Multiple features of these two signals are used for malfunction classification. The results show that the proposed method has a much better performance on classifying the normal condition and three different malfunctions in comparison with other visualization methods.

## 2. Theoretical Background

### 2.1. t-SNE Dimensionality Reduction Method

Given a high-dimensional space *X*, $X\in {\mathbb{R}}^{D}$ constitutes a data manifold for which a sample of points is available. Data points $x_{i},i=1,...,n$ in *X* are projected to points $y_{i},i=1,...,n$ in the projection space $Y\in {\mathbb{R}}^{d}\;d\;\ll \;D$ such that as much structure as possible is preserved. Stochastic Neighbor Embedding (SNE) starts by converting the high-dimensional Euclidean distances between data points into conditional probabilities that represent similarities. The similarity of data point *x_j_* to data point *x_i_* is the conditional probability, *p_j|i_*, that *x_i_* would pick *x_j_* as its neighbor if neighbors were picked in proportion to their probability density under a Gaussian centered at *x_i_*. For the nearby two data points, *p_j|i_* is relatively high, whereas for the separated data points, *p_j|i_* is low. *p_j|i_* will be almost infinitesimal, when two data points are widely separated.

In the low-dimensional space, the projected data points *y_i_* and *y_j_* can also compute a similar conditional probability, which is denoted by *q_j|i_*. SNE aims to find a low-dimensional data representation that minimizes the mismatch between *p_j|i_* and *q_j|i_*. This is achieved by minimizing a cost function which is a sum of Kullback-Leibler (KL) divergences between *p_j|i_* and *q_j|i_*. This function can be represented as:

(1) $$C=\sum_{i}KL(P_{i}\|Q_{i})=\sum_{i}\sum_{j}p_{j|i}\log\frac{p_{j|i}}{q_{j|i}}$$

Although SNE constructs a reasonably good visualization, it is hampered by a cost function that is difficult to optimize and by a problem referred to as the “crowding problem”. Van der Maaten and Hinton proposed a new technique called “t-Distributed Stochastic Neighbor Embedding” or “t-SNE” that aims to alleviate these problems [28]. t-SNE uses a symmetrized version of the SNE cost function with simpler gradients that was introduced by Cook *et al.* [30].

Instead of minimizing the sum of the KL divergences between the conditional probabilities *p_j|i_* and *q_j|i_*, it is possible to minimize a single KL divergence between a joint probability distribution, which is P in high-dimensional space and Q in low-dimensional space:

(2) $$C=KL(P\|Q)=\sum_{i}\sum_{j}p_{ij}\log\frac{p_{ij}}{q_{ij}}$$

In Equation (2), $p_{ij}=(p_{i|j}+p_{j|i})/2n$. This type of SNE is defined as symmetric SNE, because it has the property that $p_{ij}=p_{ji}$ and $q_{ij}=q_{ji}$.

Then in this symmetric SNE, the pairwise similarities in high-dimensional space is:

(3) $$p_{ij}=\frac{\exp(-{\left\|x_{i}-x_{j}\right\|}^{2}/2\sigma^{2})}{\sum_{k}\sum_{l,l\neq k}\exp(-{\left\|x_{k}-x_{l}\right\|}^{2}/2\sigma^{2})}$$

t-SNE uses a Student-t distribution to compute the similarity between two points in the low-dimensional space. t-SNE employs a heavy-tailed distribution in the low-dimensional space to alleviate both the crowding problem and the optimization problem of SNE.

In low-dimensional space, the pairwise similarities is:

(4) $$q_{ij}=\frac{{\left(1+{\left\|y_{i}-y_{j}\right\|}^{2}\right)}^{-1}}{\sum_{k}\sum_{l,l\neq k}{\left(1+{\left\|y_{k}-y_{l}\right\|}^{2}\right)}^{-1}}$$

The minimization of the cost function Equation (2) is performed by a gradient descent method. The gradient can be represented as Equation (5):

(5) $$\frac{\delta C}{\delta y_{i}}=4\sum_{j}\left(p_{ij}-q_{ij})(y_{i}-y_{j}\right){\left(1+{\left\|y_{i}-y_{j}\right\|}^{2}\right)}^{-1}$$

Then $y_{i}$ is updated by Equation (6). In order to speed up the optimization and to avoid poor local minima, a relatively large momentum term is added to the update equation:

(6) $$y_{i}^{\left(t\right)}=y_{i}^{\left(t-1\right)}+\eta \frac{\delta C}{\delta y_{i}}+\alpha (t)(y_{i}^{\left(t-1\right)}-y_{i}^{\left(t-2\right)})$$

In Equation (6), α(t) is the momentum term at iteration *t*, η is the learning rate.

### 2.2. Feature Subset Score Based t-SNE

t-SNE has good data visualization performance. It can keep the structure of different classes without class labels when reducing the dimensionality of data. In the classification problem, multiple features constitute the high-dimensional data. Not all the features are useful for classification. For example, in the malfunction classification of diesel engines, in order to reflect the engine state, many features from different measurement signals are used. Among these features, irrelevant and non-sensitive features may deteriorate the data visualization performance. Consequently, the classification accuracy is reduced. In addition, a large number of features will cause higher computational cost. If an optimal feature subset for classification can be found, the result of data visualization for classification can be effectively improved.

Suppose the original high-dimensional data space $X\in {\mathbb{R}}^{D}$ , the number of features (dimensions) is *D*. The task is to find the optimal projection matrix $W\in {\mathbb{R}}^{D\times d}$ (usually d<D) under an appropriate criterion, and the *D*-features data ***x*** from space *X* is transformed to the *d*-features data ***y*** by:

(7) $$y=W^{T}x$$

where $W\in {\mathbb{R}}^{D\times d}$ is a selection matrix. Define a column vector $w_{i}\in {\mathbb{R}}^{d}$ which has the form:

(8) $$w_{i}={\left[\underset{i-1}{\underset{︸}{0,...0,}}1,\underset{d-i}{\underset{︸}{0,...,0}}\right]}^{T}$$

Then ***W*** can be written as:

(9) $$W={\left[w_{I(1)},w_{I(2)},...,w_{I(d)}\right]}^{T}$$

where the vector ***I*** is a permutation of {*1,2,…,D*}.

A graph is a natural and effective way to encode the relationship among data. It has been applied in many machine learning tasks, such as clustering [31], subspace learning [32,33], and manifold learning [34]. For the task of feature selection, two weighted matrixes are usually constructed, $A_{w}$ and $A_{b}$. ${\text{(}A_{w})}_{ij}$ reflects the within-class or local affinity relationship between $x_{i}$ and $x_{j}$, while ${\text{(}A_{b})}_{ij}$ reflects the between-class or global affinity relationship $x_{i}$ and $x_{j}$ [35]. These two weighted matrixes are constructed according to the Fisher score method [36].

The optimal features for visualization need to satisfy $\sum_{ij}{\left\|y_{i}-y_{j}\right\|}^{2}{\left(A_{w}\right)}_{ij}$ as small as possible and $\sum_{ij}{\left\|y_{i}-y_{j}\right\|}^{2}{\left(A_{b}\right)}_{ij}$ as large as possible. To achieve the above two goals, the optimization problem can be given by:

(10) $$\psi (W)=\frac{\sum_{ij}{\left\|y_{i}-y_{j}\right\|}^{2}{\left(A_{b}\right)}_{ij}}{\sum_{ij}{\left\|y_{i}-y_{j}\right\|}^{2}{\left(A_{w}\right)}_{ij}}$$

The natural solution to this problem is to solve a trace ratio optimization problem. It can be represented by:

(11) $$\psi (W)=\frac{tr(W^{T}XL_{b}X^{T}W)}{tr(W^{T}XL_{w}X^{T}W)}$$

In Equation (11), $L_{w}$ and $L_{b}$ are the Laplacian matrices. $L_{w}=D_{w}-A_{w}$, where $D_{w}$ is a diagonal matrix, ${\left(D_{w}\right)}_{ii}=\sum_{j}{\left(A_{w}\right)}_{ij}$. $L_{b}=D_{b}-A_{b}$, where $D_{b}$ is a diagonal matrix, ${\left(D_{b}\right)}_{ii}=\sum_{j}{\left(A_{b}\right)}_{ij}$.

Define the score of a feature subset is represented as:

(12) $$score(W)=\frac{tr(W^{T}XL_{b}X^{T}W)}{tr(W^{T}XL_{w}X^{T}W)}$$

The task of feature selection is to find the feature subset with the maximum score, which can be represented by Equation (13):

(13) $$score(W_{I})=\arg\underset{W_{I}}{\max}\frac{tr(W_{I}^{T}XL_{b}X^{T}W_{I})}{tr(W_{I}^{T}XL_{w}X^{T}W_{I})}$$

A novel iterative algorithm to efficiently solve this optimization problem is proposed in literature [33].

Define $B=XL_{b}X^{T}$ and $E=XL_{w}X^{T}$:

(14) $$\lambda =\frac{tr(W^{T}BW)}{tr(W^{T}EW)}$$

For the score of the *i-th* feature can be computed by:

(15) $$score_{f}(i)=w_{i}^{T}(B-\lambda E)w_{i}$$

*d* largest scores are selected to construct $W_{I}$ according to the $score_{f}$. Then the λ is updated by Equation (14). The maximum λ can be obtained by this iterative procedure. After the convergence condition is reached, the optimal feature subset can be computed by Equation (7). The algorithm of this feature subset score based t-SNE (FSS-t-SNE) data visualization method is illustrated in Algorithm 1.

**Algorithm 1** Feature Subset Score Based t-SNE Data Visualization MethodInput: Data set $X=\{x_{1},x_{2},...,x_{n}\}$The selected feature number *d*Ouput: 2-dimensional data representation $Y=\{y_{1},y_{2},...,y_{n}\}$1. Compute the $A_{w}$ and $A_{b}$2. According to *d* random features, initialize $W_{I_{0}}$ by Equations (8) and (9), and let $\lambda_{\text{0}}=\frac{tr(W_{I_{0}}^{T}BW_{I_{0}})}{tr(W_{I_{0}}^{T}EW_{I_{0}})}$3. Compute the $score_{f}$ of each feature, $score_{f}(i)=w_{i}^{T}(B-\lambda_{n}E)w_{i}$4. Rank the features according to the $score_{f}$ in descending order5. Select the *d* features with largest scores to update $W_{I_{n}}$, and $\lambda_{n+1}=\frac{tr(W_{I_{n}}^{T}BW_{I_{n}})}{tr(W_{I_{n}}^{T}EW_{I_{n}})}$6. Iteratively perform the step 3–5 until $\left|\lambda_{n+1}-\lambda_{n}\right|<\varepsilon$7. Compute new data set $X^{*}=\{x_{1}^{*},x_{2}^{*},...,x_{n}^{*}\}$ by $x_{i}^{*}=W_{I}^{T}x_{i}$8. Initialize $Y_{0}=\{y_{1},y_{2},...,y_{n}\}$ from $N(0,10^{-4})$9. Compute conditional probabilities $p_{j|i}$ and $p_{i|j}$ from $X^{*}$, set $p_{ij}=(p_{i|j}+p_{j|i})/2n$10. Compute $q_{ij}$ from $Y_{n}$11. Compute gradient $\frac{\delta C}{\delta Y}$12. Set $Y_{n+1}=Y_{n}+\eta \frac{\delta C}{\delta Y}+\alpha (t)(Y_{n}-Y_{n-1})$13. Iteratively perform the step (10–12) until preset iteration steps.14. 2-dimensional data $Y=\{y_{1},y_{2},...,y_{n}\}$ obtained.

## 3. UCI Dataset Test for the FSS-t-SNE Method Validation

The dataset from the UCI machine learning repository are used for validating the proposed method [37]. We choose “Australian Sign Language signs (High Quality)” dataset from this repository. The samples of this dataset were captured from a native signer using high-quality position trackers [38]. This dataset is usually used for classification task. In this test, four different signs are selected for data visualization and classification. These four signs are “change mind”, “answer”, “forget” and “deaf”, which are represented by Sign1, Sign2, Sign3 and Sign4, respectively. The original features of this dataset are 22. The features are constituted of pattern data recorded for each hand, such as palm positions, palm degrees, finger bend measures, *etc*. The more information can be obtained from the website of UCI machine learning repository.

**Figure 1:**
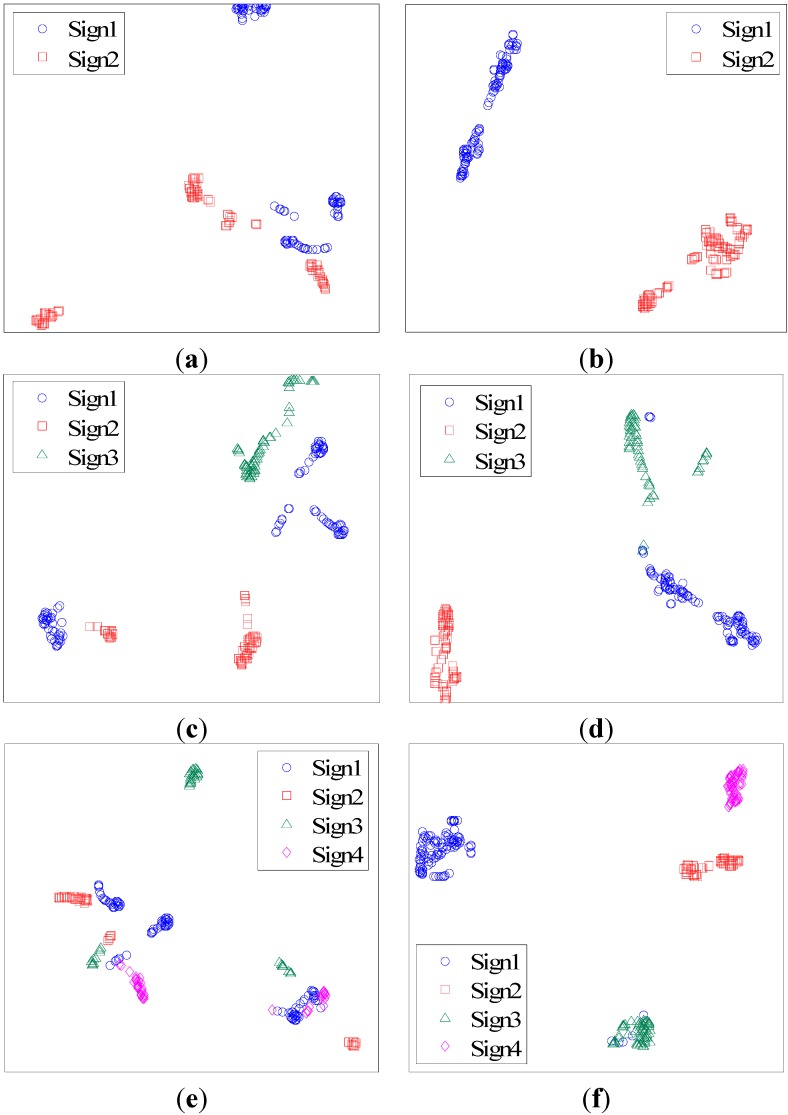
(**a**) t-SNE for two signs; (**b**) FSS-t-SNE for two signs; (**c**) t-SNE for three signs; (**d**) FSS-t-SNE for three signs; (**e**) t-SNE for four signs; (**f**) FSS-t-SNE for four signs.

The number of features in optimal feature subset is set to 4. As FSS-t-SNE is a proposed method based on t-SNE, the results of the t-SNE method are used for comparison. The data visualization results are shown in Figure 1. Figure 1a,b show the visualization results of two signs. In Figure 1a, it is hard to find a dividing line between Sign 1 and Sign 2. In Figure 1b, the two signs can be easily separated. Figure 1c,d show the results of three signs. Figure 1e,f show the results of four signs.

According to the results, FSS-t-SNE can improve the data visualization performance for classification. In order to evaluate the improvement for classification, the k-Nearest Neighbor (KNN) classifier is used for classifying the different signs. In KNN classifier, the number of the neighbors is set to 1. Ten samples of each sign are used for training the classifier. The results of classification accuracy are shown in Table 1. It is shown that FSS-t-SNE can improve the classification accuracy effectively.

**Table 1:** Classification accuracy by KNN classifier.

Item	t-SNE	FSS-t-SNE
2 Signs	50.88%	100%
3 Signs	50.73%	98.53%
4 Signs	53.56%	85.77%

## 4. Data Visualization for Malfunction Classification on Diesel Engine

### 4.1. Feature Set Construction from Multi-Sensor Signals

**Figure 2:**
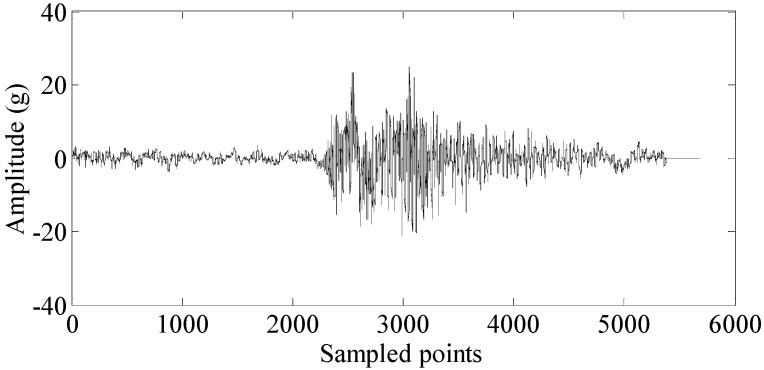
Combustion segment from a cylinder vibration signal.

**Figure 3:**
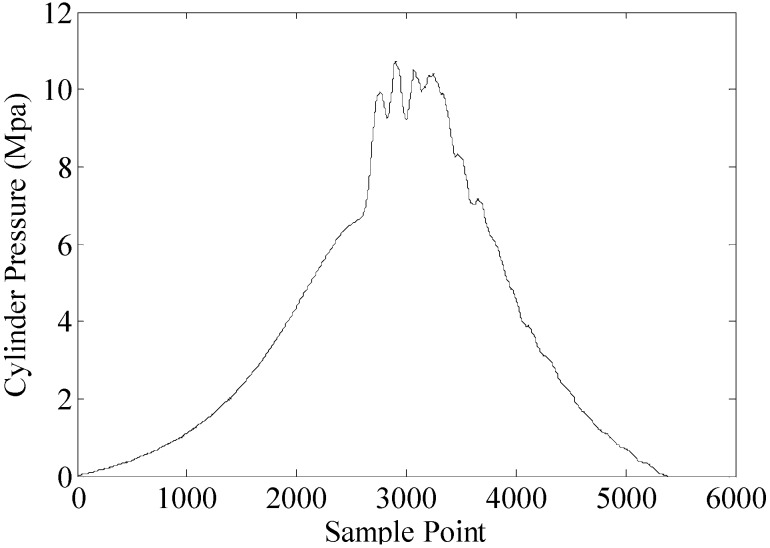
Combustion segment from a cylinder pressure signal.

The combustion process is an important part in a working cycle of the diesel engine. Most malfunctions of diesel engines can be reflected in the combustion process. For instance, when a fault occurs on the fuel pump of a cylinder, it causes fuel supply problems, and the combustion of the corresponding cylinder is inevitably weaker. As mentioned in Section 1, cylinder vibration and pressure are mostly used in the condition monitoring of diesel engine. Therefore, the combustion segment is drawn from these two signals to analyze the different malfunctions of diesel engines. Figure 2 shows the combustion segment from the cylinder vibration signal. Figure 3 shows the combustion segment from the cylinder pressure signal.

The combustion of the diesel engine is a very intricate process. It is hardly possible to classify the different malfunctions by a single feature. Therefore, more features should be extracted to construct a feature set. According to the figure, the combustion segments from the two measurement methods are both non-stationary signals. In general, when malfunctions occur, this can be reflected in the time-domain parameters of the signals. In this paper, 15 time-domain features are considered. They are described in Table 2.

**Table 2:** The 15 time-domain features.

Feature	Equation
Root Mean Square	$p_{1}=\sqrt{\frac{1}{N}\sum_{l=1}^{N}x_{i}^{2}}$
Square Root Amplitude	$p_{2}={\left(\frac{1}{N}\sum_{l=1}^{N}\sqrt{\left|x_{l}\right|}\right)}^{2}$
Absolute Average	$p_{3}=\frac{1}{N}\sum_{l=1}^{N}\left|x_{l}\right|$
Skewness	$p_{4}=\frac{1}{N}\sum_{l=1}^{N}{\left|x_{l}\right|}^{3}$
Kurtosis	$p_{5}=\frac{1}{N}\sum_{l=1}^{N}x_{l}^{4}$
Variance	$p_{6}=\frac{1}{N}\sum_{l=1}^{N}x_{l}^{2}$
Maximum	$p_{7}=\max(x_{l})$
Minimum	$p_{8}=\min(x_{l})$
Peak to Peak Value	$p_{9}=p_{7}-p_{8}$
Waveform Index	$p_{10}=\frac{p_{1}}{p_{3}}$
Crest Index	$p_{11}=\frac{p_{7}}{p_{1}}$
Pulse Index	$p_{12}=\frac{p_{7}}{p_{3}}$
Abundance Index	$p_{13}=\frac{p_{7}}{p_{2}}$
Skewness Index	$p_{14}=\frac{p_{4}}{p_{6}^{3/2}}$
Kurtosis Index	$p_{15}=\frac{p_{5}}{p_{6}^{2}}$

**Figure 4:**
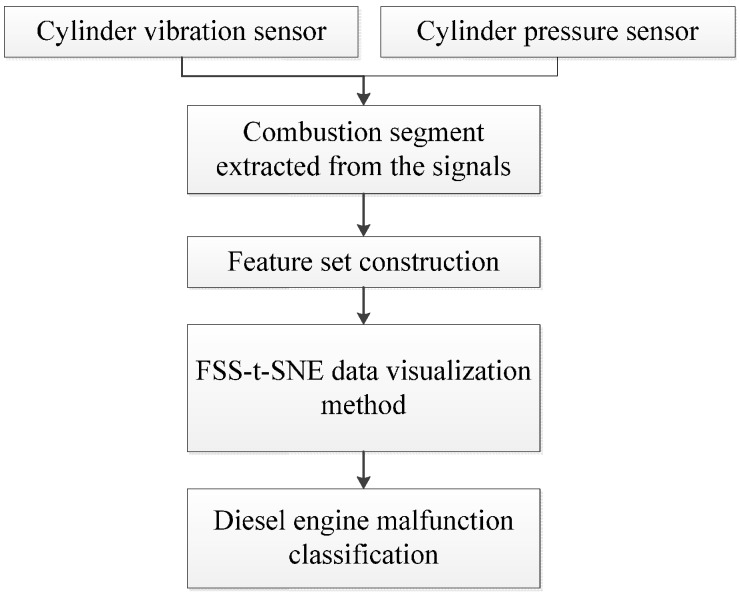
The procedure of the malfunction classification on diesel engines.

For each measurement method, the original signal and its envelope signal are used to compute the time-domain features. Then, a feature set with 60 dimensions is constructed. This high-dimensional data is used as the input of the proposed data visualization method. According to the result of the data visualization, classification task is performed by a classifier. The procedure of the malfunction classification on a diesel engine can be represented by a flowchart that is shown in Figure 4.

### 4.2. Diesel Engine Malfunction Experiment

**Figure 5:**
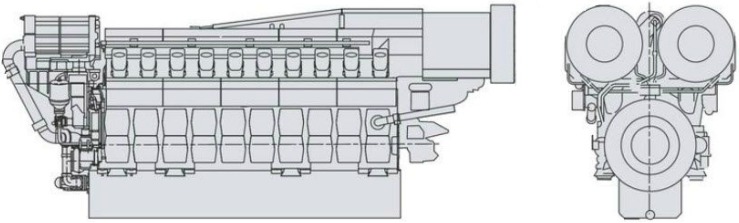
Large power marine diesel engine.

In order to validate the proposed method applied on malfunction classification of a diesel engine, a diesel engine malfunction experiment was performed. In this experiment, a large power marine diesel engine, which was being inspected before shipment was used. A drawing of this diesel engine is shown in Figure 5.

**Figure 6:**
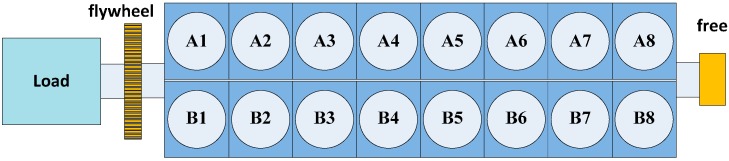
Structure of the diesel engine.

**Figure 7:**
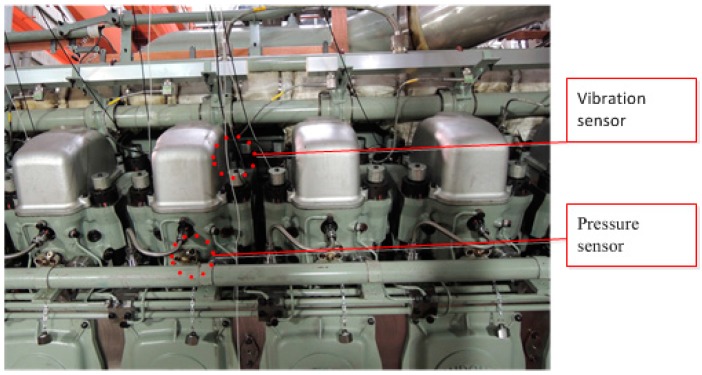
Setup view of cylinder A5.

The basic technical data of this diesel engine is shown in Table 3. The structural representation of this engine is shown in Figure 6. There are 16 cylinders in this diesel engine. They are arranged in two banks, which are named as Bank A and Bank B, respectively. The malfunctions were created on cylinder A5. Figure 7 shows the setup view of cylinder A5. The vibration sensor was mounted on top of the cylinder. The pressure sensor was mounted on the screwed hole on the top of the cylinder.

**Table 3:** Engine technical specifications.

Item	Content
Number of Cylinders	16
Cylinder arrangement	Dual V type
Cycle	4 stroke
Induction System	Turbocharge
Stroke × Bore	330 mm × 280 mm
Firing Order	A1-B1-A6-B6-A2-B2-A4-B4-A8-B8-A3-B3-A7-B7-A5-B5
Gross Power	5700 kW@1084 rpm

The specifications of the malfunctions created in this experiment are described in Table 4. Because the combustion process of diesel engine is affected by many parameters, such as the engine load, engine speed, engine age, exact fuel injection time, *etc*., the cylinder pressure signals and cylinder vibration signals may vary when parameters change. In order to reduce the effect of engine age, a diesel engine which was being inspected before shipment was used to perform the bench experiment. In this bench experiment, the parameters such as engine load, engine speed, fuel injection time, fuel condition, *etc.* were controlled under the same conditions. All the malfunctions were created individually. Three different malfunction conditions and normal working condition were performed in this experiment. To create a lack of fuel supply, the fuel supply controller was adjusted to supply low fuel. To create bad fuel atomization, a faulty fuel atomizer nozzle was replaced on the corresponding cylinder. To create an exhaust valve leakage, a hole was milled on the exhaust valve of the corresponding cylinder. Under each condition, the engine was run at 835 ± 3 rpm. When the engine was running in stationary state, signal sampling was performed.

**Table 4:** Malfunction experiment specifications.

Engine Condition	Fault Cylinder	Implementation Method
Normal Condition	-	-
Lack of Fuel Supplying	A5	The deflation valve of induction system is opened.
Bad Fuel Atomization	A5	A faulty fuel atomizer nozzle is replaced on the corresponding cylinder.
Leakage of Exhaust Valve	A5	A hole is milled on the exhaust valve of the corresponding cylinder.

**Figure 8:**
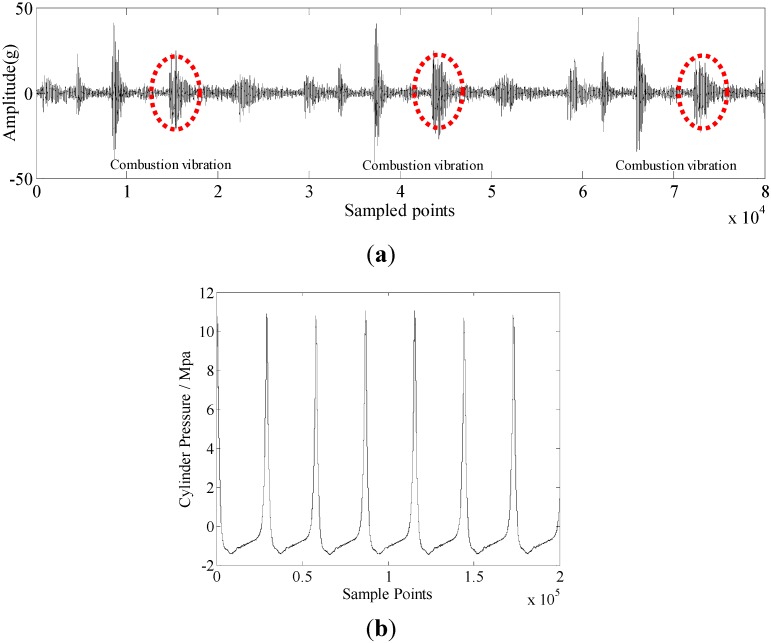
(**a**) Sampled cylinder vibration signal; (**b**) Sampled cylinder pressure signal.

Cylinder vibration and pressure signals were sampled from cylinder A5. Figure 8a shows a segment of the sampled cylinder vibration signal. There are many vibration sources in the sampled vibration data, including intake valve vibrations, exhaust valve vibrations, fuel pump vibrations, combustion vibrations, *etc*. The combustion vibration is used to analyze the different conditions of the diesel engine. The red circles in Figure 8a indicate the combustion vibrations. Figure 8b shows a segment of the sampled cylinder pressure signal.

## 5. Results and Discussion

**Figure 9:**
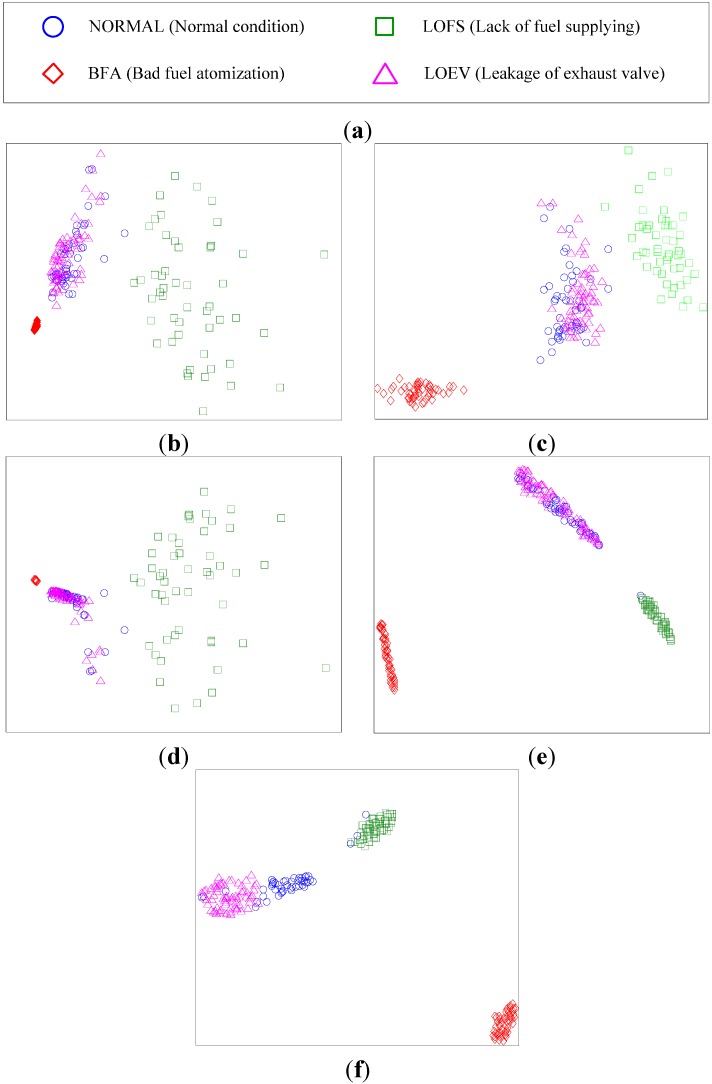
(**a**) Symbols description; (**b**) PCA; (**c**) LDA; (**d**) ISOMAP; (**e**) t-SNE; (**f**) FSS-t-SNE.

The cylinder vibration and pressure signals were sampled under four different conditions in the malfunction experiment, including normal condition and three malfunction conditions. Different features are extracted from these signals. The high-dimensional data of the four conditions are used by the data visualization method for classification. The FSS-t-SNE method is compared with other methods, including linear methods such as PCA and LDA, and non-linear methods such as ISOMAP and t-SNE. The data visualization results are shown in Figure 9.

The linear methods, PCA and LDA, which are shown in Figure 9b,c, can only show three different clusters. In Figure 9b, the LOFS condition doesn’t have a good clustering performance. Compared with the PCA method, the LDA method shown in Figure 9c has better clustering performance. However, in both figures, the NORMAL condition and the LOEV condition are completely overlapped. It is impossible to separate these two conditions by the two methods. The non-linear methods, ISOMAP and t-SNE, are shown in Figure 9d,e. They can also show the three different clusters. Compared with the two linear methods above, the ISOMAP method is more similar to the PCA method. t-SNE has the best performance among these methods. In Figure 9e, the points belonging to the same class is closely conglomerated, whereas those belonging to different classes are clearly separated, except for the NORMAL and LOEV conditions. Apparently, it still cannot separate the NORMAL and LOEV conditions of the diesel engine. In Figure 9e, the NORMAL and LOEV conditions are still completely overlapped. Figure 9f shows the result of the proposed FSS-t-SNE method. It clearly shows four different clusters. Although the NORMAL and LOEV conditions are close to each other, they don’t overlap and can be separated easily. Moreover, the good clustering performance of the t-SNE method is retained.

**Figure 10:**
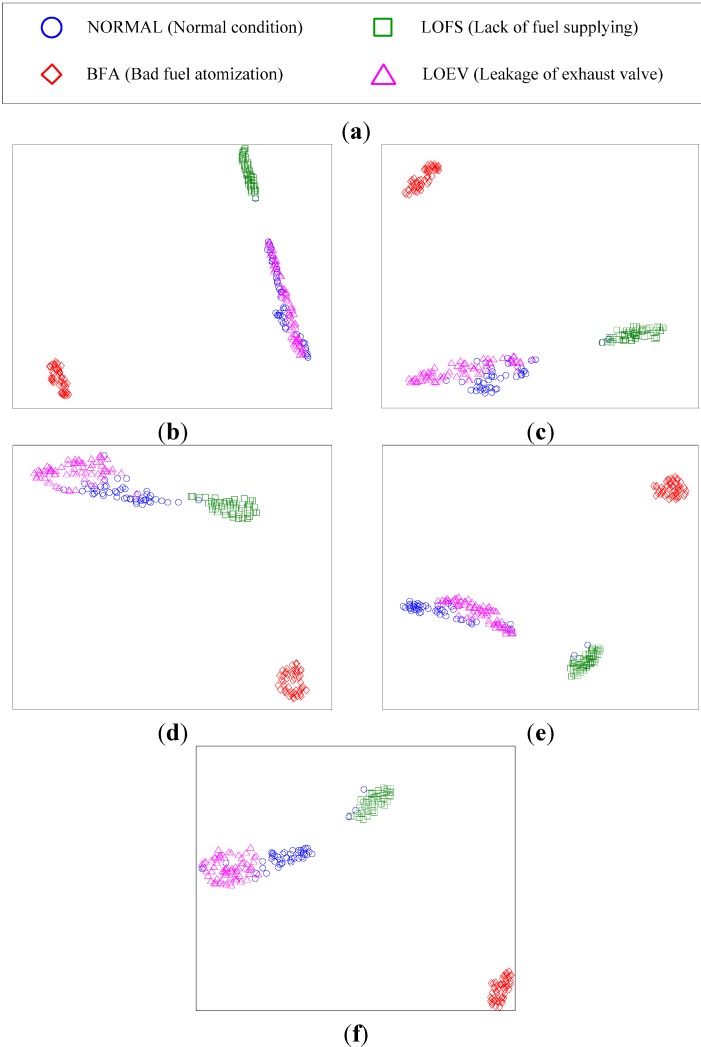
(**a**) Symbols description; (**b**) 55 features; (**c**) 45 features; (**d**) 30 features; (**e**) 15 features; (**f**) 10 features.

Figure 9 demonstrates that the proposed method, FSS-t-SNE, outperforms the other methods in data visualization of diesel engine multi-sensor data under different conditions. In the proposed FSS-t-SNE method, the number of features contained in the optimal feature subset is a variable parameter. According to the principle of feature selection, different numbers of features form different optimal feature subsets. They have different performance in data visualization. Figure 10 shows the data visualization results of different optimal feature subsets that contains different numbers of features selected by FSS-t-SNE. When 55 features are selected, it is nearly the same to the result of t-SNE shown in Figure 9e.

**Figure 11:**
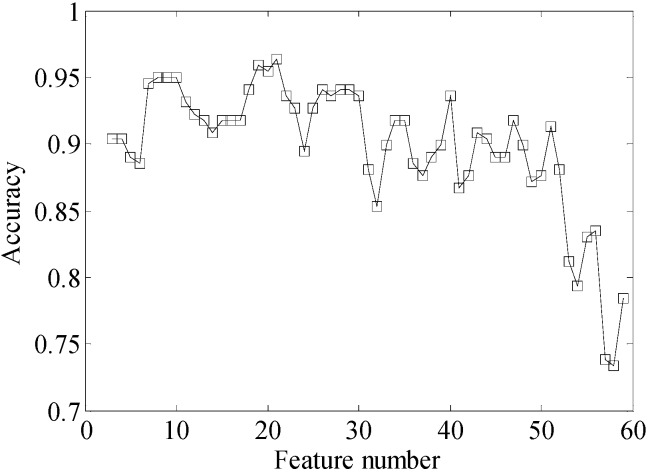
Classification accuracy of different feature number.

The NORMAL and LOEV conditions are completely overlapped. As the number of features decreases, the overlapped two classes are separated gradually. The clustering performance is also improved. From Figure 10e, it can be seen that the two overlapped classes are separated when 10 features are selected. In order to analyze the improvement of classification performance when selected features change, the KNN classifier is used to compute the classification accuracy of four conditions. In KNN classifier, the number of the neighbors is set to 1. Ten samples of each condition of the diesel engine are used for training the KNN classifier. The accuracy curve is shown in Figure 11.

According to the accuracy curve, the FSS-t-SNE has high accuracy when the number of selected features is less than 50. The accuracy is higher than 85% when the selected number is less than 50. When 20 features are selected, the malfunction classification has the highest accuracy. The accuracy decreases after the number of features is less than 10. As shown in the Figure 11, the accuracy curve is not smooth. It fluctuates when the number of features changes. There are two main causes for this result. First, FSS-t-SNE initializes the low-dimensional data with the random data points. Selecting different features may lead to different distribution results of the data points that belong to the same class. However, the samples used for training the KNN classifier are the same. This may have an effect on the classification accuracy of the KNN classifier. Second, the samples for computing the classification accuracy are limited. Because the aim of our study is a large power marine diesel engine, in order to reduce the effect of engine age, we used a brand new engine which was being inspected before shipment. The malfunction experiment may cause damage to a new engine. Therefore, the engine could only be run for a short time under each malfunction condition and limited samples were collected from the engine. Although the accuracy curve fluctuates, the whole tendency of the curve shows that the classification accuracy is improved.

## 6. Conclusions

A diesel engine is a complex dynamic machine. It is difficult to evaluate the state of the engine by single features extracted from the single measurement signal. In order to solve this problem, various features extracted from multi-sensor signals are used to identify and classify the different states or malfunctions of the engine. These different features from multi-sensor signals constitute a high-dimensional dataset. The identification and classification problem is transformed to solve the pattern recognition problem with this high-dimensional data. Data visualization using the dimensionality reduction method is an effective way. In the malfunction classification of diesel engines, not all the features are sensitive to the specific malfunction condition. Some features may deteriorate the result of classification. Irrelevant and non-sensitive features should be eliminated before data visualization. For this purpose, a feature subset score based t-SNE data visualization method is proposed in this paper. Optimal feature subset is obtained by the feature subset score criterion. Then the non-linear dimensionality reduction method t-SNE is used for data visualization. Malfunction experiments with a large power diesel engine were performed to validate the proposed method. According to the experimental results, the proposed method has high classification accuracy in malfunction classification of the diesel engine.

## References

[1] Watzenig D., Sommer M.S., Steiner G. (2013). Model-Based Condition and State Monitoring of Large Marine Diesel Engines.

[2] Angelov P., Giglio V., Guardiola C., Lughofer E., Lujan J.M. (2006). An approach to model-based fault detection in industrial measurement systems with application to engine test benches. Meas. Sci. Technol..

[3] Serdio F., Lughofer E., Pichler K., Buchegger T., Pichler M., Efendic H. (2014). Fault detection in multi-sensor networks based on multivariate time-series models and orthogonal transformations. Inf. Fusion.

[4] Chandroth G.O., Sharkey A.J.C., Sharkey N.E. (1999). Cylinder Pressures and Vibration in Internal Combustion Engine Condition Monitoring.

[5] Sharkey A.J.C., Chandroth G.O., Sharkey N.E. Acoustic emission, cylinder pressure and vibration: A multisensor approach to robust fault diagnosis. Proceedings of the IEEE-INNS-ENNS International Joint Conference on Neural Networks. IJCNN 2000. Neural Computing: New Challenges and Perspectives for the New Millennium.

[6] Geng Z., Chen J. (2005). Investigation into piston-slap-induced vibration for engine condition simulation and monitoring. J. Sound Vib..

[7] Porteiro J., Collazo J., Patino D., Miguez J.L. (2011). Diesel engine condition monitoring using a multi-net neural network system with nonintrusive sensors. Appl. Therm. Eng..

[8] Yang J.G., Pu L.J., Wang Z.H., Zhou Y.C., Yan X.P. (2001). Fault detection in a diesel engine by analysing the instantaneous angular speed. Mech. Syst. Signal Process..

[9] Taglialatela F., Lavorgna M., Mancaruso E., Vaglieco B.M. (2013). Determination of combustion parameters using engine crankshaft speed. Mech. Syst. Signal Process..

[10] Lin T.R., Tan A.C., Ma L., Mathew J. (2014). Estimating the loading condition of a diesel engine using instantaneous angular speed analysis. Engineering Asset Management 2011.

[11] Hargis S.C., Taylor H.F., Gozzo J.S. (1983). Condition monitoring of marine diesel engines through ferrographic oil analysis. Wear.

[12] Elamin F., Fan Y., Gu F., Ball A. (2009). Detection of diesel engine valve clearance by acoustic emission. Proceedings of Computing and Engineering Annual Researchers' Conference.

[13] Lin T.R., Tan A.C., Mathew J. (2011). Condition monitoring and diagnosis of injector faults in a diesel engine using in-cylinder pressure and acoustic emission techniques. Dyanmics Sustain. Eng..

[14] Lapuerta M., Armas O., Hernandez J.J. (1999). Diagnosis of di diesel combustion from in-cylinder pressure signal by estimation of mean thermodynamic properties of the gas. Appl. Therm. Eng..

[15] Payri F., Broatch A., Tormos B., Marant V. (2005). New methodology for in-cylinder pressure analysis in direct injection diesel engines-application to combustion noise. Meas. Sci. Technol..

[16] Watzenig D., Sommer M.S., Steiner G. (2009). Engine state monitoring and fault diagnosis of large marine diesel engines. Elektrotech. Inf. Tech..

[17] Padhye N., Zuo L., Mohan C.K., Varshney P.K. (2009). Dynamic and Evolutionary Multi-Objective Optimization for Sensor Selection in Sensor Networks for Target Tracking.

[18] Rajagopalan R., Mohan C.K., Varshney P., Mehrotra K. Multi-objective mobile agent routing in wireless sensor networks. Proceedings of the 2005 IEEE Congress on Evolutionary Computation.

[19] Masazade E., Rajagopalan R., Varshney P.K., Mohan C.K., Sendur G.K., Keskinoz M. (2010). A multiobjective optimization approach to obtain decision thresholds for distributed detection in wireless sensor networks. IEEE Trans. Syst. Man Cybern. Part B Cybern..

[20] Padhye N., Mohan C., Mehrotra K., Varshney P. Sensor selection strategies for networks monitoring toxic chemical release. Proceedings of the ISCA First International Conference on Sensor Networks and Applications.

[21] Rodger J.A. (2012). Toward reducing failure risk in an integrated vehicle health maintenance system: A fuzzy multi-sensor data fusion kalman filter approach for ivhms. Expert Syst. Appl..

[22] Ward M.O., Grinstein G., Keim D. (2010). Interactive Data Visualization: Foundations, Techniques, and Applications.

[23] Tenenbaum J.B., de Silva V., Langford J.C. (2000). A global geometric framework for nonlinear dimensionality reduction. Science.

[24] Roweis S.T., Saul L.K. (2000). Nonlinear dimensionality reduction by locally linear embedding. Science.

[25] Kambhatla N., Leen T.K. (1997). Dimension reduction by local principal component analysis. Neural Comput..

[26] Fisher R.A. (1936). The use of multiple measurements in taxonomic problems. Ann. Eugen..

[27] Sharma A., Paliwal K.K., Onwubolu G.C. (2006). Class-dependent pca, mdc and lda: A combined classifier for pattern classification. Pattern Recognit..

[28] Van der Maaten L., Hinton G. (2008). Visualizing data using t-SNE. J. Mach. Learn. Res..

[29] Cao L.J., Chua K.S., Chong W.K., Lee H.P., Gu Q.M. (2003). A comparison of pca, kpca and ica for dimensionality reduction in support vector machine. Neurocomputing.

[30] Cook J., Sutskever I., Mnih A., Hinton G.E. Visualizing similarity data with a mixture of maps. Proceedings of the International Conference on Artificial Intelligence and Statistics.

[31] Shi J.B., Malik J. (2000). Normalized cuts and image segmentation. IEEE Trans. Pattern Anal. Mach. Intell..

[32] He X., Cai D., Niyogi P. (2005). Laplacian Score for Feature Selection.

[33] Nie F., Xiang S., Jia Y., Zhang C., Yan S. (2008). Trace Ratio Criterion for Feature Selection.

[34] Jin X.H., Zhao M.B., Chow T.W.S., Pecht M. (2014). Motor bearing fault diagnosis using trace ratio linear discriminant analysis. IEEE Trans. Ind. Electron..

[35] Lughofer E. (2011). On-line incremental feature weighting in evolving fuzzy classifiers. Fuzzy Sets Syst..

[36] Bishop C.M. (1995). Neural Networks for Pattern Recognition.

[37] Uci machine learning repository. http://archive.ics.uci.edu/ml.

[38] Kadous M.W. (2002). Temporal Classification: Extending the Classification Paradigm to Multivariate Time Series.

